# Evaluation of the Treatment Effect of *Aloe vera* Fermentation in Burn Injury Healing Using a Rat Model

**DOI:** 10.1155/2019/2020858

**Published:** 2019-01-27

**Authors:** Zhiwen Hai, Yimeng Ren, Jiawen Hu, Huan Wang, Qi Qin, Tingtao Chen

**Affiliations:** ^1^Institute of Translational Medicine, Nanchang University, Nanchang, Jiangxi 330031, China; ^2^Harbin Meihua Biotechnology Limited by Share Ltd., Harbin, Heilongjiang 150018, China

## Abstract

Burn injury is a growing medical problem associated with public health, and few effective agents are available for treatment of this disease. In the present study, a burn injury rat model was developed and the accelerated effect of *Aloe vera* fermentation on burn injury healing was evaluated. Our results indicated that *Aloe vera* fermentation could markedly reduce the DPPH (56.12%), O^2·−^ (93.5%), ^·^OH (76.12%), Fe^2+^ chelation (82%), and oxygen-reduction activity (0.28 *μ*g/ml) and significantly inhibited the growth of pathogens *S. typhimurium* ATCC 13311 (inhibition zone diameter: 14 mm), *S. enteritidis* ATCC13076 (IZD: 13 mm), *S. flexneri* ATCC 12022 (IZD: 18 mm), *E. coli* 44102 (IZD: 10 mm), *L. monocytogenes* ATCC 19111 (IZD: 18 mm), *S. dysenteriae* 301 (IZD: 20 mm), *S. aureus* COWAN1 (IZD: 19 mm), and *P. acnes* ATCC 11827 (IZD: 25 mm) *in vitro*. The *in vivo* results indicated that *Aloe vera* fermentation produced more eosinophils and fibroblasts and less vessel proliferation compared with the model group on the 14^th^ day, which had greatly accelerated burn injury healing via shedding of the scab and promoting hair growth. ELISA results indicated that *Aloe vera* fermentation had significantly reduced the production of proinflammatory factors TNF-*α* and IL-1*β* (*p* < 0.05) and greatly enhanced the yield of anti-inflammatory factor IL-4 in animal serum (*p* < 0.05). In addition, the high-throughput sequencing results indicated that *Aloe vera* fermentation obviously increased the percentage of Firmicutes (65.86% vs. 49.76%), while reducing the number of Bacteroidetes (27.60% vs. 45.15%) compared with the M group at the phylum level. At the genus level, *Aloe vera* fermentation increased the probiotic bacteria *Lactobacillus* (3.13% vs. 2.09%) and reduced the pathogens *Prevotella* (10.60% vs.18.24%) and *Blautia* (2.91% vs. 16.41%) compared with the M group. Therefore, we concluded that the use of *Aloe vera* fermentation significantly accelerates burn injury healing via reduction of the severity of inflammation and through modification of gut microbiota.

## 1. Introduction

As one of the most common severe injuries in the world, burn injury is defined as damage to the body's tissues caused by heat, chemicals, electricity, sunlight, or radiation [1]. Till now, burn injury is still challenging for clinical treatment and severe burn injury has caused a high postburn morbidity. In 2015, 67 million injuries caused by fire and heat resulted in 176,000 deaths [2, 3].

Based on depth, burns are divided into 4 types: superficial (1st degree), deep partial thickness (2nd degree), full thickness (3rd degree), and 4th degree [4]. Burns are usually preventable, and different treatments are applied based on the severity of the burn. Sometimes, ointments, creams, biological and nonbiological dressings, and antibiotics are recommended in 2nd-degree, 3rd-degree, and 4th-degree burns, while the abuse of these drugs increases the risk of antibiotic resistance and fungal infections, even slowing wound healing and increasing the depth of the burn [5, 6]. In addition, alternative medicines, e.g., honey, *Aloe vera*, oatmeal, eggs, mud, leaves, or cow dung, have been used for treatment of burns [7, 8].

*Aloe vera* originates from the Arabian Peninsula and has been planted around the world for agricultural and medicinal uses, including beverages, skin lotion, cosmetics, or ointments for minor burns and sunburns [9, 10]. Previous researches indicated that *Aloe vera* could promote wound healing in various animal models, its sound antimicrobial, antioxidative, and anti-inflammation effects contributed to local treatment of wounds, minor burns, and skin irritation [10–12]. In addition, the substances bradykinin and thromboxane found in *Aloe vera* were useful to relieve pain and promoted wound healing via promoting the fall-off of dead cells [12].

Probiotics are microorganisms that “when administered in adequate amounts, confer a health benefit on the host” [13]. Probiotics have been proven to confer health benefits via improving lactose intolerance and increasing natural resistance to infectious diseases [14–16]. As one of the famous probiotics, *Lactobacillus plantarum* has been isolated from various fermented foods and host intestine, and this strain grows well in harsh conditions and inhibits the pathogenic growth of *Shigella dysenteriae*, *Staphylococcus aureus*, *Enterobacter sakazakii*, and *Escherichia coli* [14].

In our previous study, *L. plantarum* had been used for the fermentation of *Aloe vera*, and the *Aloe Vera* fermentation showed sound effects of antioxidative activity, antibacterial activity, and anti-inflammatory *in vivo* [17]. In the present study, we constructed a burn injury model and evaluated the accelerated effects of *Aloe vera* fermentation on burn injury healing.

## 2. Materials and Methods

### 2.1. *Aloe vera* Fermentation

The bottom of *Aloe vera* leaves were cut off and washed thoroughly, then their edges were removed and the slimy and transparent gel contents were obtained via peeling leaves. A pulper was used to mash the gel, which was then centrifuged at 5000 ×g for 20 min to obtain the *Aloe vera* supernatant.

*L. plantarum* HM218749 was cultured using de Man–Rogosa–Sharpe (MRS) medium at 37°C for 24 h, then *L. plantarum* HM218749 was added into the *Aloe vera* extract + 5% glucose to reach the final concentration of 5 × 10^6^ cfu/ml, which was then cultured for another 48 h. *Aloe vera* fermentation was obtained via centrifugation of the fermented supernatant at 5000 ×g for 20 min.

### 2.2. Antioxidative and Antibacterial Activity

The *Aloe vera* fermentation was used to test its antioxidative ability by the oxygen-reduction activity of DPPH, O_2_^·−^, ^·^OH, Fe^2+^ chelation, and oxygen reduction, and the *Aloe vera* supernatant was used as the control. To test the antimicrobial ability of the *Aloe vera* fermentation and *Aloe vera* supernatant, the Oxford cup method was used to evaluate the inhibition effect of *Aloe vera* fermentation and *Aloe vera* supernatant on the pathogens *Salmonella typhimurium* ATCC 13311, *S. enteritidis* ATCC13076, *S. flexneri* ATCC 12022, *E. coli* 44102, *Listeria monocytogenes* ATCC 19111, *S. dysenteriae* 301, *S. aureus* COWAN1, and *Propionibacterium acnes* ATCC 11827. The experiment was carried out in duplicate [18].

### 2.3. Animal Model of Burn Injury

Eight-week-old female rats, provided by the animal experiment center of Nanchang University (Nanchang, Jiangxi, China), were housed in the animal facility under standard conditions (humidity 50 ± 15%, temperature 22 ± 2°C, 12/12 light-dark cycle) and fed a standard diet. Nembutal (40 mg/kg) was intraperitoneally (i.p.) injected into the rats, and the hairs were removed using a clipper. Anesthetized rats were placed on a plastic template and received scald injuries using the iron which was immersed in a 100°C water bath.

Animals were randomly divided into three groups (*n* = 5 per group): the model group (M), resuscitated with 1.0 ml of 0.9% normal saline; the *Aloe vera* fermentation group (AFB), resuscitated with 1.0 ml of *Aloe vera* fermentation; and the burn cream group (BC), resuscitated with 1.0 ml of burn cream (Bovec skin burn cream, no. YDH007923, Bunker Industrial Co., Ltd., China, Nanchang, China). A control group (C) was set up.

### 2.4. HE Stain

According to the manufacturer's protocol, the skin tissue was cut according to the edge of the wound, and all the injured tissues were cut off and were fixed in 4% (*v*/*v*) paraformaldehyde. All samples were stained with haematoxylin and eosin (HE) [19].

### 2.5. ELISA

Blood samples were collected from each rat. Then blood samples were centrifuged at 1500 g. Serum was stored at −80°C until it was analyzed. According to the manufacturer's protocol, we measured the concentration of TNF-*α*, IL-1*β*, and IL-4 by using an ELISA kit for TNF-*α* (eBioscience), IL-1*β* (eBioscience), and IL-4 (eBioscience) [20].

### 2.6. DNA Extraction and High-Throughput Sequencing Analyses

Tree rats in each group were randomly chosen, and their fresh fecal samples were collected for DNA extraction using a genome DNA Kit (TIANGEN Biotechnology Co., Ltd., Beijing, China).

The concentration and quality of the extracted genomic DNA were tested before using a nanoparticle spectrophotometer for sequencing [21]. Using the extracted genomic DNA as a template, 338 F/806R primers were used to amplify the V3-V4 region of the 16S rRNA gene, the PCR reaction, the pyrophosphoric acid sequencing of the PCR amplification products, and the quality control of the original data. The paired end readings of the original DNA fragment using Flash, when at least some reads overlap from the relative end of the same DNA fragment, were merged into the paired end read, and the paired end reads were allocated to each sample. Then, the UPARSE-OTU and UPARSE-OTUref algorithms were used to analyze the UPARSE package. Internal Perl scripts were used to analyze the diversity of *α* (intrasample) and *β* (between samples). The same OTU was assigned a sequence with more than 97% similarity. The selection sequence was represented by each OTU, and the RDP classifier was used to annotate the categorization information of each representative sequence. The QIIME software package was used to weigh the clustering results and found that the diversity of species had the metabolic ability of PICRISE (1.00.0) (GenBank accession number SRP155691) [22].

### 2.7. Bacterial Counts

Bacterial counts were performed based on previous reports [16]. Plates with 30–300 colonies were replica-plated onto de Man–Rogosa–Sharpe (MRS) agar (*Lactobacillus*), Slanetz–Bartley medium (SBM) agar (*Enterococci*), and MacConkey agar (*Enterobacteria*) and were then incubated aerobically at 37°C for 24 to 36 h for colony counting.

### 2.8. Data Analysis

Statistical analyses were performed with SPSS 30 software (SPSS, Chicago, IL). *p* values < 0.05 were considered as statistically significant.

## 3. Result

### 3.1. Antioxidative and Antibacterial Activity of *Aloe vera* Fermentation *In Vitro*

**Figure 1:**
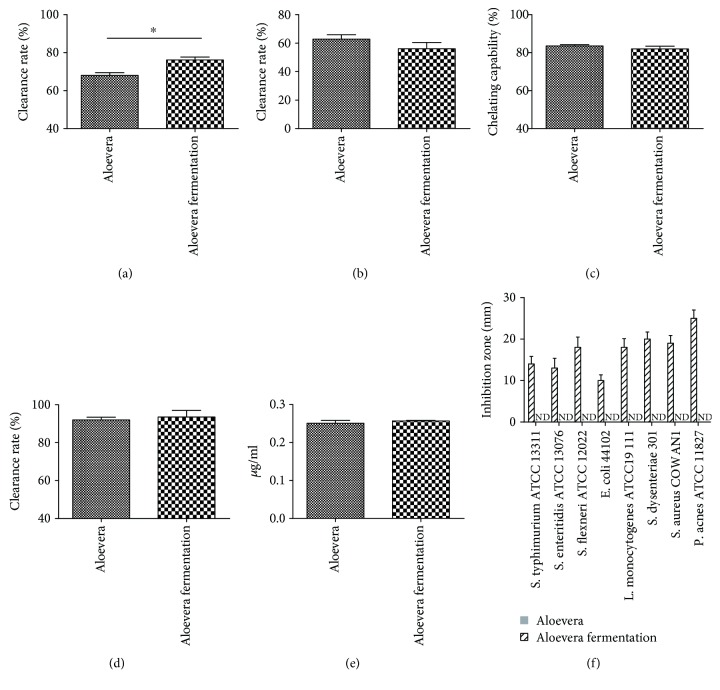
The antioxidant effect of *Aloe vera* and *Aloe vera* + *L. plantarum* are shown in the clearance rate of OH^−^ (a), clearance rate of DPPH (b), chelation rate of Fe^2+^ (c), clearance rate of O_2_^−^ (d), and the total reducing power (e). The antimicrobial activity of the *Aloe vera* and *Aloe vera* + *L. plantarum* against the pathogens which have been selected and cultured in MRS is shown in (f). Data are shown as the mean ± SD. ^∗^ *p* < 0.05.

Firstly, we evaluated the antioxidant and antibacterial activities of *Aloe vera* fermentation. The results indicated that *Aloe vera* fermentation had significantly enhanced the clearance rate of ^·^OH compared with *Aloe vera* (76.12% vs. 68.00%, Figure 1(a), *p* < 0.05), while no marked difference was observed among their capabilities with regard to the clearance rate of DPPH (56.12% vs. 62.83%, Figure 1(b)), chelation rate of Fe^2+^ (82.00% vs. 83.50%, Figure 1(c)), clearance rate of O_2_^·−^ (93.50% vs. 92.00%, Figure 1(d)), and the total reducing power (0.26 *μ*g/ml vs. 0.25 *μ*g/ml, Figure 1(e)). Similar to our previous results, no antimicrobial effect was observed in the *Aloe vera* group, while *Aloe vera* fermentation showed a perfect inhibition effect on the growth of *S. typhimurium* ATCC 13311 (inhibition zone diameter: 14 mm), *S. enteritidis* ATCC13076 (IZD: 13 mm), *S. flexneri* ATCC 12022 (IZD: 18 mm), *E. coli* 44102 (IZD: 10 mm), *L. monocytogenes* ATCC 19111 (IZD: 18 mm), *S. dysenteriae* 301 (IZD: 20 mm), *S. aureus* COWAN1 (IZD: 19 mm), and *P. acnes* ATCC 11827 (IZD: 25 mm) (Figure 1(f)).

### 3.2. Using of *Aloe vera* Fermentation Accelerated Healing of Burn Injury

**Figure 2:**
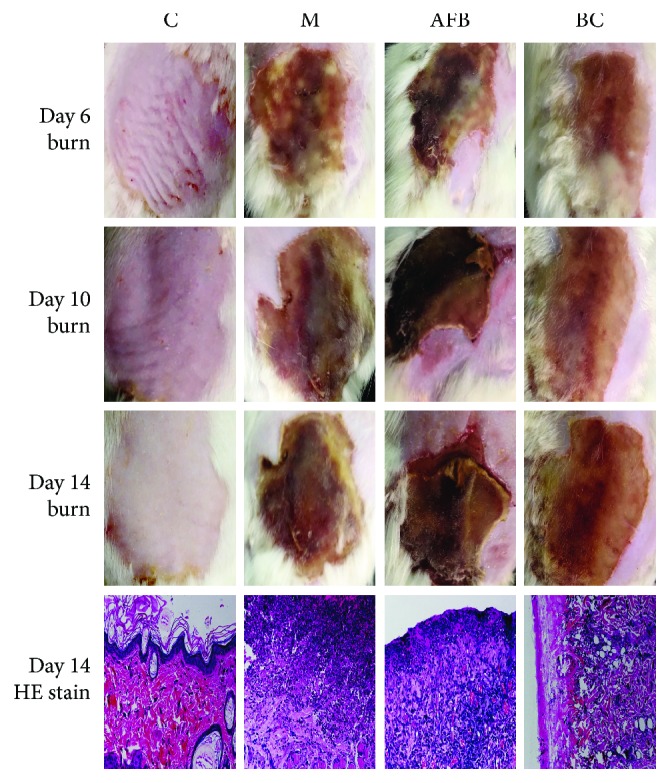
Treatment of the Aloe-fermented liquid on burn rats using morphology evaluation (days 6, 10, and 14) and histological evaluation (day 14). C, control group; M, model group; AFB: aloe-fermented liquid group; BC: burn cream group.

Daily administration of *Aloe vera* fermentation and burn cream (BC group, positive control) was carried out. The photos of the wound on days 6, 10, and 14 are presented in Figure 2, and their hair, shedding of scab, and color of the wounds were compared. In the M group, although new hairs were growing in wounds, some green purulent fluid (caused by inflammation) still existed from day 6 to day 14; in the AFB group, the shedding of the scab appeared in these wounds, and new hairs were growing well. Though the burn cream group showed a better effect on wound healing than the M group, no scab was shed and the wound still possessed a white color compared with the AFB group (Figure 2).

On day 14, HE results indicated that cells in the C group were intact; the stratum corneum, epidermis, and dermal structure were clear; and the hair follicle and skin accessory structures were intact. Compared with the M group and the BC group, more eosinophils and fibroblasts appeared in wound tissues and less vessel proliferation were observed, indicating that *Aloe vera* fermentation had a better effect to accelerate the wound to normal levels (Figure 2).

### 3.3. The Anti-Inflammatory Activity of *Aloe vera* Fermentation

**Figure 3:**
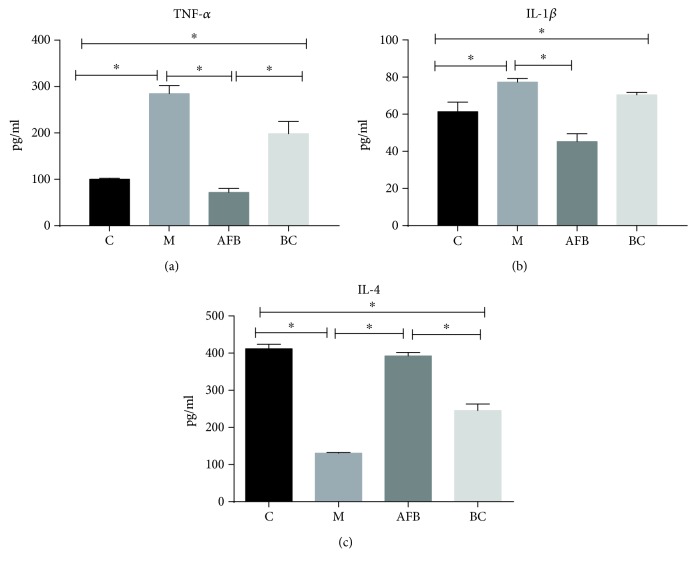
Effect of the aloe-fermented liquid on the production of inflammatory TNF-*α* (a), IL-1*β* (b), and IL-4 (c) in the blood using the ELISA method. C, control group; M, model group; AFB: aloe-fermented liquid group; BC: burn cream group. Data are shown as the mean ± SD. ^∗^ *p* < 0.05.

Then, we checked the yields of inflammatory factors (TNF-*α*, IL-1*β*, and IL-4) in rat serum. As shown in Figure 3, the pathology of burn injury had significantly enhanced the production of the proinflammatory factors TNF-*α* (284.82 pg/ml vs. 100.14 pg/ml) and IL-1*β* (77.32 pg/ml vs. 61.38 pg/ml), while markedly reducing the yield of the anti-inflammatory factor IL-4 (130.68 pg/ml vs. 411.71 pg/ml) compared with the control group (*p* < 0.05). The use of *Aloe vera* fermentation had significantly converted the harmful effect of the burn injury via reduction of TNF-*α* (71.70 pg/ml vs. 284.82 pg/ml) and IL-1*β* (45.29 pg/ml vs. 77.32 pg/ml) and enhanced IL-4 (392.19 pg/ml vs. 130.68 pg/ml) compared with the M group. Although the burn cream group presented a sound effect on anti-inflammatory activity, it showed a weaker effect on reducing the productions of TNF-*α* (198.51 pg/ml vs. 71.70 pg/ml) (*p* < 0.05) and IL-1*β* (70.48 pg/ml vs. 45.29 pg/ml) and an enhanced effect on IL-4 (245.63 pg/ml vs. 392.19 pg/ml) (*p* < 0.05) compared with the AFB group (Figure 3).

### 3.4. Compositions and Relative Abundance of Bacterial Communities in the Gut

**Figure 4:**
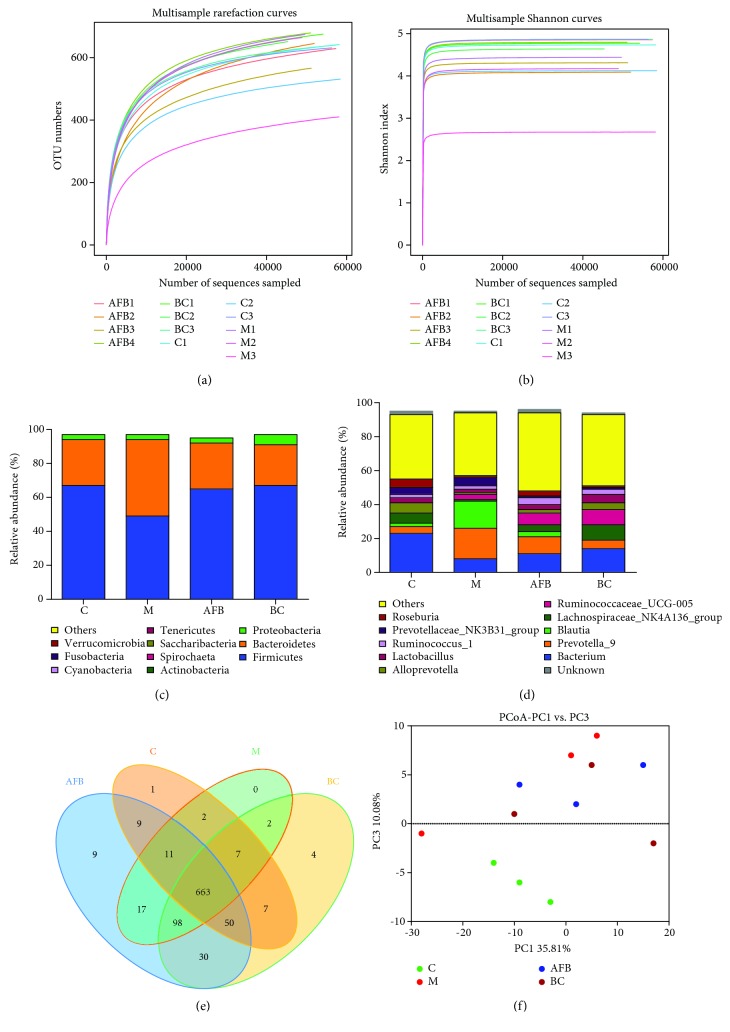
Evaluation of the aloe-fermented liquid on intestinal microbiota of burn rats using high-throughput sequencing: (a, b) the Shannon and rarefaction index among groups C, M, AFB, and BC; (c) the relative abundance of the bacteria among groups C, M, AFB, and BC at the phylum level; (d) the relative abundance of the bacteria among groups C, M, AFB, and BC at the genus level; (e) Scalar-Venn representation of the intestinal microbiota among the C, M, AFB, and BC groups; (f) the PCoA analysis of the C, M, AFB, and BC groups. C, control group; M, model group; AFB: aloe-fermented liquid group; BC: burn cream group.

As the burn injury, together with the inflammation caused by wounds, would alter the microbial diversity in rat intestines, therefore we monitored the microbial population among groups C, M, AFB, and BC using the high-throughput sequencing method. In total, 780,118 clean tags (60,009.07 tags/sample) and 8037 OTUs were obtained from all samples with an average of 618.23 OTUs per group (Table S1), and the rarefaction curve and Shannon curves were saturated and every sample in all groups entered the plateau phase (Figures 4(a) and 4(b)).

At the phylum level, burn injury had obviously reduced the percentage of Firmicutes (49.76% vs. 67.96%), while increasing the number of Bacteroidetes (45.15% vs. 27.67%), while use of *Aloe vera* fermentation and burn cream reversed this trend (Figure 4(c)). At the genus level, the overgrowth of the pathogens *Prevotella* (18.25% vs. 3.99%) and *Blautia* (16.41% vs. 2.70%) and the reduced percentage of probiotic bacteria of *Lactobacillus* (2.09% vs. 3.30%) compared with the control group indicated that burn injury had disrupted the intestinal microbial balance, while the increased number of *Lactobacillus* (3.13% vs. 2.09%), and reduced number of *Prevotella* (10.6% vs. 18.25%) and *Blautia* (2.91% vs. 16.41%), compared with the M group, indicated that the use of *Aloe vera* fermentation was useful to restore the microbial balance to normal levels (Figure 4(d)).

In addition, the Venn method indicated that 663 common OTUs were found, which occupied 88.4% (663/750), 82.88% (663/800), 80.17% (663/827), and 77.00% (663/861) in the C, M, AFB, and BC groups, respectively (Figure 4(e)). The PCoA analysis also indicated that the samples in the M group departed far away from the C group, and samples in the AFB and BC groups had a closer distance to the C group compared with the samples in the M group (Figure 4(f)).

### 3.5. Effect of *Aloe vera* Fermentation on Intestinal Microbial Number

**Figure 5:**
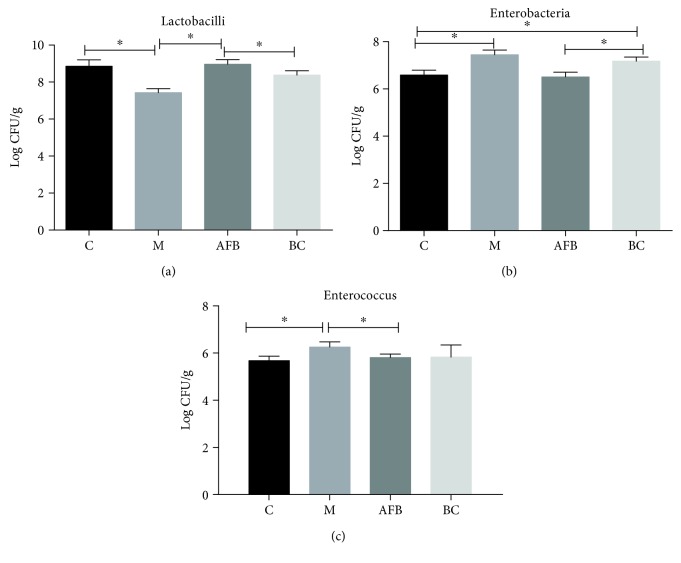
The fecal microbial number of genus *Lactobacillus*, *Enterobacteriaceae*, and *Enterococcus* among the C, M, AFB, and BC groups using the viable cell count method. C, control group; M, model group; AFB: aloe-fermented liquid group; BC: burn cream group. Data are shown as the mean ± SD. ^∗^ *p* < 0.05.

In the end, we counted the microbial number using a viable cell counting method. As shown in Figure 5, the burn injury had significantly reduced the number of *Lactobacilli* (7.43 CFU/g vs. 8.86 CFU/g) and made an overgrowth of the pathogens *Enterobacteria* (7.45 CFU/g vs. 6.59 CFU/g) and *Enterococcus* (6.26 CFU/g vs. 5.68 CFU/g), and the use of *Aloe vera* fermentation and burn cream was beneficial for the microbiota to resume to their normal levels.

## 4. Discussion

Burns are the fourth leading cause of trauma in road traffic accidents [23]. After the burn, the wound was sterile almost for 24-48 hours, and the Gram-positive organism will form a sweat gland which delays the separation of eschar and the growth of healthy granulation tissue, prolonging the length of stay and increasing the threat [24].

For burn injury, wound healing and infection prevention were important for patients. Therefore, the sound promotion effect of wound healing of *Aloe vera* and the sound antibacterial effect of probiotics via yielding of antibacterial metabolites made *Aloe vera* fermentation a perfect choice for burn injury [25, 26]. At first, we tested the antioxidative and antibacterial activity of *Aloe vera* fermentation *in vitro* and found that *Aloe vera* fermentation possessed a sound antioxidative activity via reduction of the DPPH, O_2_^·−^, ^·^OH, Fe^2+^ chelation, and oxygen-reduction activity, and this fermentation had inhibited all the test pathogens we used (Figure 1). Therefore, the antioxidant capacity and antibacterial capacity of *Aloe vera* fermentation showed its potential as a burn injury agent. Oxidation is a chemical reaction to produce free radicals, which leads to the chain reactions and damage of the cells of the host. The oxidative stress contributes to the development of various diseases, e.g., diabetes, neurodegeneration, fat, and cancer [27, 28]. Moreover, the sound antibacterial activity of *Aloe vera* fermentation showed that this agent can effectively inhibit/kill most common pathogens around the patients to avoid bacterial infections.

Then, a burn injury rat model was developed, and *Aloe vera* fermentation had greatly accelerated burn injury healing via shedding of the scab and promoting the growth of hair (Figure 1). The HE stain also confirmed that *Aloe vera* fermentation had produced more eosinophils, more fibroblasts, and less vessel proliferation compared with the M group (Figure 2). As we know, the main healing cells of hair sweat glands and follicles were responsible for the healing of the epithelial healing phase, and their appearance was beneficial to the migration of the epithelial cells in the new tissue to form scars [29, 30]. During the proliferation of fibroblasts and epithelial cells, blood vessels were formed to supply nutrition of reactive oxygen at the initial stage and decreased at the final phase of healing, which is beneficial to form the hydrated matrix to promote cell migration and wound healing [31].

During wound healing, immune cells (macrophages, mast cells, or damaged fibroblasts) are accumulated in wound tissues, which stimulated the release of proinflammatory mediators [32]. Therefore, we tested the concentration of proinflammatory factors TNF-*α*, IL-1*β*, and the anti-inflammatory factor IL-4 in an animal serum. Just as we guessed, *Aloe vera* fermentation had significantly reduced TNF-*α* and IL-1*β* and enhanced IL-4 compared with the M group (Figure 3, *p* < 0.05). Previous studies show that elevated levels of TNF-*α* and IL-1 might be an important factor in the occurrence of multiple organ failure after thermal injury; therefore, the increase of the TNF-*α* and IL-1*β* circulating levels after a burn indicated a poor prognosis [33–35]. For IL-4, this cytokine can activate the M2 cell to repair wounds and inhibit the expression of TNF-*α* and IL-1, playing a role in anti-inflammation [36].

In the end, we monitored the microbial diversity using the high-throughput sequencing method and the viable cell counting method. Our results indicated that *Aloe vera* fermentation had obviously increased the percentage of Firmicutes (65.86% vs. 49.76%), while reducing the number of Bacteroidetes (27.67% vs. 45.15%) compared with the M group (Figure 4(c)). Moreover, *Aloe vera* fermentation did increase the probiotic bacteria *Lactobacillus* and reduced the pathogens *Prevotella* and *Blautia* compared with the M group. Previous studies indicated that *Lactobacillus* constituted a significant component in the digestive system, urinary system, and genital system, and its metabolites could effectively inhibit a variety of pathogens and sustain homeostasis of the host intestine [37]. *Prevotella* is a genus of Gram-negative bacteria and often causes infections including inhalation pneumonia, lung abscess, pulmonary empyema, chronic otitis media, and sinusitis [38]. Moreover, the viable cell counting method also confirmed that use of *Aloe vera* fermentation had significantly increased the number of *Lactobacilli* and reduced the pathogens *Enterobacteria* and *Enterococcus* (Figure 5).

In the present study, *Aloe vera* fermentation presented a sound effect of antioxidant and antibacterial activities, and it can significantly accelerate healing of a burn injury via reduction of the inflammatory condition and microbial disorders caused by the burn injury. Therefore, this agent maybe potentially used as a burn injury drug to accelerate the healing process and guard human health. However, the sound healing effects of *Aloe vera* fermentation are mainly based on animal experimentation; therefore, more work is need to optimize *Aloe vera* fermentation, enhance its efficacy on burn injuries, and verify its effectiveness in the clinic.

## Data Availability

The raw sequencing data of the high-throughput sequencing used to support the findings of this study are included within the article.

## Supplementary Materials

Supplementary Materials Table S1: the number of raw tags, clean tags, effective tag AvgLen, and OTUs in groups C, M, 393, AFB, and BC by high-throughput sequencing.
