# Supplementary material for: Evaluation of the Treatment Effect of Aloe vera Fermentation in Burn Injury Healing Using a Rat Model
Source: Mediators Inflamm. 2019 Jan 27;2019:2020858. doi: 10.1155/2019/2020858 (PMC6374857; doi:10.1155/2019/2020858)
Supplement: Supplementary Materials — Table S1: the number of raw tags, clean tags, effective tag AvgLen, and OTUs in groups C, M, 393, AFB, and BC by high-throughput sequencing. [file 2020858.f1.pdf]

Table S1 Number of raw tags, clean tags, effective tags AvgLen and OTUs in groups C, M AFB and BC by high-throughput sequencing.

Sample name	Raw Tags	Clean Tags	Effective Tags	AvgLen(bp)	OTU_Num
AFB1	70663	59603	59457	415	629
AFB2	70424	59510	57916	417	645
AFB3	71670	61396	59010	413	566
AFB4	71349	60523	59519	414	679
BC1	70497	59358	58706	415	675
BC2	71869	61554	59130	412	651
BC3	71001	60067	59976	416	640
C1	71638	60634	60432	415	642
C2	71183	60309	60242	413	531
C3	70980	60583	60350	412	631
M1	70342	58341	56998	418	674
M2	70050	58304	57296	418	664
M3	71206	59936	59414	415	410
Total	994542	780118	768446	5393	8037
Average	76503.23	60009.07	59111.23	414.84	618.23
